# Use of genetics in the prediction of success in male pattern hair loss therapy and mechanistic studies

**DOI:** 10.3389/fphar.2026.1765808

**Published:** 2026-02-12

**Authors:** Gustavo Torres de Souza, Greg Williams, Carolina Costa Vicente Silva, Caroline Brandão Chiovatto, Gorana Kuka Epstein, Laura Vila-Vecilla, Valentina Russo

**Affiliations:** 1 Laboratory of Genetics, Fagron Genomics, Research and Development Department, Barcelona, Spain; 2 Farjo Hair Institute, London, United Kingdom; 3 Fagron BV, Research and Development Department, Rotterdam, Netherlands; 4 Fagron Brasil, São Paulo, Brazil; 5 Foundation for Hair Restoration, Miami, FL, United States

**Keywords:** androgenetic alopecia, genetics, hair loss, minoxidil, pharmacogenetics

## Abstract

Male pattern hair loss, the clinical manifestation of androgenetic alopecia in men, is a highly prevalent chronic condition associated with significant psychosocial burden, yet current therapies show heterogeneous efficacy and tolerability between individuals. Over the past decade, genome wide association and sequencing studies have identified hundreds of susceptibility loci that converge on androgen signalling, WNT pathways, prostaglandin metabolism, extracellular matrix remodelling, vascular regulation, telomere biology, and cellular metabolism, indicating that male pattern hair loss is mechanistically tractable and strongly genetically determined. In parallel, pharmacogenetic work has linked variants in genes involved in minoxidil bioactivation, 5α-reductase isoenzyme activity, prostaglandin synthesis, collagen organisation, and vascular tone to differences in treatment response. In this narrative review, we integrate evidence from large genetic studies, targeted pharmacogenetic cohorts, transcriptomic and pathway analyses, and preclinical models to delineate how genetic architecture informs disease mechanisms and modulates the effects of established therapies such as topical and oral minoxidil, finasteride, dutasteride, and prostaglandin-directed approaches. We also discuss emerging targets, including IGF1R, WNT10A, PPARGC1A, and prolactin receptor signalling, and examine how RNA based androgen receptor silencing and stem-cell-derived regenerative strategies exploit these pathways. Together, these data support a shift from empirical prescribing towards genetically informed, mechanism anchored treatment algorithms for male pattern hair loss, in which pharmacogenetic markers, polygenic scores, and multi omic readouts are progressively incorporated into therapeutic decision making and the design of future clinical trials.

## 1 Introduction

Androgenetic alopecia (AGA) is the most common form of progressive scalp hair loss in men, characterised clinically by patterned miniaturisation of follicles in the frontal, mid-scalp, and vertex regions, with relatively preserved occipital and parietal hair growth. A well-established epidemiological pattern shows that approximately 30% of men exhibit clinically relevant AGA by the age of 30, rising to nearly 50% by age 50, and exceeding 70% in later decades of life (Liu et al., 2025b). The condition typically manifests after puberty, with prevalence increaseing steadily with age and reaching the majority of men (Nestor et al., 2021; Liu et al., 2025b). Although often labelled as a cosmetic problem, AGA is associated with substantial psychosocial burden, including impaired self-esteem, body image disturbance, and symptoms of anxiety and depression, which justify its consideration as a chronic medical condition that benefits from early diagnosis, sustained non-surgical management, and hair transplant surgery (Nestor et al., 2021; Aukerman and Jafferany, 2023; Liu et al., 2025b; Williams, 2025). Epidemiological studies further indicate variation in age at onset, severity, and pattern across ancestries, indicating interfering factors associated to the pathogenesis (Liu et al., 2025b). In men, androgenetic alopecia manifests clinically as male pattern hair loss (MPHL), and this review focuses on the genetics, mechanisms, and treatment response of MPHL.

Family clustering and twin studies have long suggested a strong inherited component in AGA in men, and recent large genome-wide association studies have expanded this understanding into a detailed polygenic architecture with more than 380 susceptibility loci identified to date (Heilmann-Heimbach et al., 2017; Liu et al., 2025b). These loci include variants in and around the androgen receptor (AR) gene, genes involved in androgen metabolism, WNT signalling components, and regulators of extracellular matrix, inflammation, and vascular tone, illustrating that AGA reflects a complex interplay between androgen signalling and broader hair follicle regulatory networks (Li et al., 2012; Heilmann-Heimbach et al., 2017). Genetic studies of early-onset AGA have also revealed shared susceptibility loci with cardiometabolic and autoimmune traits, suggesting pleiotropic effects that may partly explain reported associations between AGA, metabolic syndrome, and cardiovascular risk (Su and Chen, 2010; Li et al., 2012; Bakry et al., 2014; Liu et al., 2025b). At the follicular level, mechanistic work links this inherited background to androgen-driven changes in dermal papilla signalling, oxidative stress, premature cellular senescence, and altered epithelial–mesenchymal crosstalk, which together favour progressive follicle miniaturisation and shortening of the anagen phase (Leirós et al., 2012; Upton et al., 2015; Chew et al., 2016; Dhurat and Daruwalla, 2021).

Current pharmacological management of male pattern hair loss relies primarily on minoxidil and 5α-reductase inhibitors, complemented by procedural and regenerative approaches. Topical minoxidil remains a first-line therapy and promotes hair growth through vasodilatory and mitogenic effects, although its precise mechanisms and inter-individual variability in response are still being refined (Messenger and Rundegren, 2004; Suchonwanit et al., 2019; Liu et al., 2025b). Oral low-dose minoxidil has emerged as an increasingly used alternative, particularly in patients who have difficulty adhering to topical regimens or in whom systemic exposure is preferred, albeit with a distinct systemic safety profile (Chen et al., 2025; Liu et al., 2025b). In men, finasteride and dutasteride reduce dihydrotestosterone levels by inhibiting type II or both type I and type II 5α-reductase respectively. Multiple long-term studies and integrated evidence syntheses demonstrate their efficacy in stabilising or improving AGA, with dutasteride showing higher efficacy in some comparisons but also raising concerns about sexual and neuropsychiatric adverse effects (Liu et al., 2025b). Additional options include topical formulations of 5α-reductase inhibitors, low-level light therapy, platelet-rich plasma (PRP), microneedling, and ultimately hair transplantation, yet real-world outcomes remain heterogeneous and often fall short of patient expectations (Nestor et al., 2021; Shrestha et al., 2024; Chen et al., 2025).

In parallel, an expanding pipeline of emerging therapies seeks to address key molecular pathways and regenerative deficits that are insufficiently targeted by conventional drugs. These include small-molecule activators or stabilisers of WNT/β-catenin signalling, inhibitors of prostaglandin D_2_ signalling, prostaglandin F_2_α analogues, modulators of JAK–STAT and PDE pathways, and novel agents aimed at mitochondrial and metabolic regulation of follicle cycling (Garza et al., 2012; Bellani et al., 2025; Zhi et al., 2025). Cell-based and extracellular vesicle-based therapies, such as mesenchymal stem cell-derived exosomes or dermal papilla cell products, have shown promising effects on hair follicle activation, angiogenesis, and anagen induction in preclinical and early clinical studies, and are being explored alone or in combination with PRP and microneedling as part of regenerative protocols (Rajendran et al., 2017; Ersan et al., 2024; Chen et al., 2025). Furthermore, liposomal mRNA delivery, antibody therapies, and bioengineering of new follicles represent conceptually distinct approaches that aim to reprogramme or replace miniaturised follicles rather than simply slowing their decline (Chen et al., 2025; Feng et al., 2025; Liu et al., 2025b, 2025a). Although many of these strategies remain experimental, they underscore a shift from empiric to mechanism-based development in AGA.

Against this background of complex biology and expanding treatment options, inter-individual variability in efficacy and tolerability has become a central challenge. Patients differ markedly in their response to minoxidil, finasteride, dutasteride, and regenerative interventions, even when disease stage and treatment duration are comparable, which suggests that genetic variation in drug targets, metabolic enzymes, signalling pathways, and tissue microenvironment is an important but under-used determinant of outcome (Garza et al., 2012; Suchonwanit et al., 2019; Chen et al., 2025; Liu et al., 2025b). Our recent work using pharmacogenetic panels has started to link specific single-nucleotide polymorphisms (SNPs) in genes such as SULT1A1, SRD5A1, SRD5A2, PTGES2, and PTGFR to differential responses to minoxidil and 5α-reductase inhibitors, and to patterns of network activation in prostaglandin, collagen, and inflammatory pathways, laying the groundwork for genotype-informed treatment selection (de Souza, 2025; Gaboardi et al., 2025). The purpose of this review is to build on these advances by integrating current genetic and pharmacogenetic evidence with mechanistic insights into AGA pathophysiology and drug response, and to discuss how genomic profiling might be deployed to predict therapeutic success, refine mechanisms of action for existing and emerging therapies, and support the development of truly personalised, mechanism-anchored treatment strategies in androgenetic alopecia in men (Li et al., 2012; Heilmann-Heimbach et al., 2017; de Souza, 2025; Liu et al., 2025b) (Box 1).

BOX 1 **Linking biological targets to realistic clinical outcomes in male pattern hair loss.**

**Biological targets in androgenetic alopecia are mechanistically coherent but clinically constrained.**

Current pharmacological and regenerative therapies for male pattern hair loss act on defined biological levers, including androgen signalling, follicular cycling programmes, prostaglandin balance, vascular support, and the perifollicular microenvironment. Genetic and pharmacogenetic studies increasingly clarify which pathways are perturbed in individual patients. However, the clinical effects achievable by targeting these pathways are inherently limited by disease stage, tissue context, and compensatory biological processes.

**Target intent and expected clinical endpoints.**

Interventions that reduce androgen-driven catagen pressure, such as 5α-reductase inhibitors or local androgen receptor suppression, are primarily expected to stabilise disease progression and permit partial recovery of miniaturised follicles, rather than fully restore terminal hair density. Agents that support anagen initiation or prolongation, including minoxidil and prostaglandin F_2_α analogues, may increase hair shaft calibre or density modestly, but rarely reverse advanced miniaturisation. Regenerative or microenvironment-focused approaches aim to improve vascular supply, extracellular matrix quality, and inflammatory tone, with the goal of preserving follicular viability rather than recreating normal follicle architecture.

**Why responses are partial, heterogeneous, or plateauing.**

Even when biological targets are appropriately engaged, outcomes frequently plateau or vary between individuals. This reflects several converging mechanisms already discussed in this review, including shortened anagen competence, progressive perifollicular fibrosis, altered extracellular matrix remodelling, oxidative stress, cellular senescence, and pathway redundancy within the follicular niche. As miniaturisation advances towards irreversible atrophy, the capacity for meaningful structural recovery diminishes despite continued pathway modulation.

**Implications for combination and multi-target strategies.**

These constraints support a shift away from single-pathway expectations towards rational, non-competing combination approaches. Targeting androgen signalling, follicular cycling, and the microenvironment in parallel may reduce non-response and delay plateauing by addressing distinct biological bottlenecks rather than intensifying a single mechanism. In this context, genetic and pharmacogenetic profiling may assist in prioritising which biological levers are most relevant for a given patient, while realistic clinical endpoints remain stabilisation, modest regrowth, or delayed progression rather than complete follicular restoration.

## 2 Molecular basis of hair follicle physiology and androgenetic alopecia

Family, twin, and segregation studies consistently show that AGA, particularly male-pattern hair loss (MPHL), is a highly heritable trait, with twin- and family-based heritability estimates frequently approaching or exceeding 80%, providing a strong rationale for systematic genetic mapping and downstream functional studies (Heilmann-Heimbach et al., 2017; Yap et al., 2018b, 2018a). Large genome-wide association studies (GWAS) in European cohorts have identified dozens of loci associated with MPHL, with early meta-analyses reporting 63 genome-wide significant regions that together explained about 39% of the variance in liability, already indicating a relatively tractable polygenic architecture compared with many other complex traits (Heilmann-Heimbach et al., 2017). More recent analyses integrating UK Biobank data expanded this architecture into the hundreds of near-independent loci and reported SNP-based heritability estimates around 0.4, whereas pedigree-based estimates derived from first-degree relatives remained higher, close to 0.6, reflecting the additional contribution of rare, structural, or regulatory variants that are not captured by common SNPs. (Pickrell et al., 2016; Henne et al., 2023). Recent integrative reviews summarise several hundred non-redundant risk regions for AGA or MPHL, many of which map to genes involved in androgen signalling, WNT-driven morphogenesis, and follicle cycling, supporting a model in which numerous small-effect variants converge on a limited set of biological modules in the hair follicle microenvironment (Henne et al., 2023; Liu et al., 2025b). Together, these data establish that common SNPs account for a substantial proportion of heritable susceptibility, while leaving room for rarer coding variants and regulatory changes that are only now being resolved through exome and whole-genome sequencing (Yap et al., 2018b, 2018a; Henne et al., 2023).

Across these GWAS and follow-up studies, several loci have emerged as particularly robust and mechanistically informative. The X-chromosomal region around the AR and ectodysplasin A2 receptor (EDA2R) was the first major genetic signal, and remains the single strongest contributor to MPHL risk, consistent with the strict androgen dependence of the phenotype and the central role of dermal papilla androgen signalling in hair follicle miniaturisation (Li et al., 2012; Heilmann-Heimbach et al., 2017; Henne et al., 2023). Independent autosomal loci at chromosome 20p11, identified in multiple studies, further contribute substantially to risk, although the exact causal genes and mechanisms at this region remain under investigation (Hillmer et al., 2008; Henne et al., 2023). Additional loci harbour genes involved in androgen metabolism, such as SRD5A1 and SRD5A2, as well as CYP19A1, which together modulate dihydrotestosterone (DHT) production and local androgen balance in the scalp (Li et al., 2012; Heilmann-Heimbach et al., 2017). A second major group of signals maps to WNT and related morphogenetic pathways, with strong evidence for WNT10A, RSPO2, LGR4, DKK2, and FGF5, all of which influence anagen induction, hair shaft formation, and follicle size (Heilmann et al., 2013; Heilmann-Heimbach et al., 2017; Henne et al., 2023). More recent targeted and panel-based association studies have added variants in genes linked to prostaglandin synthesis and signalling (PTGES2, PTGFR, PTGDR2), collagen and extracellular matrix (COL1A1), vascular regulation (ACE), growth factor signalling (IGF1R), and retinoid binding (CRABP2), collectively reinforcing the view that androgen sensitivity, follicle cycling, lipid mediators, extracellular matrix organisation, and microvascular supply are all genetically modulated in AGA (Li et al., 2012; Păun et al., 2023; Francès et al., 2024). Within this broader WNT signalling framework, it is important to distinguish between pathway-level candidate genes identified through enrichment and functional studies, and a smaller subset of core replicated loci, notably WNT10A and WNT6, which consistently emerge across large-scale GWAS and pharmacogenetic analyses and therefore carry greater weight for genetic risk stratification and translational targeting (Li et al., 2012).

Vitamin D receptor (VDR) signalling represents a modulatory axis in hair follicle biology that intersects functionally with canonical WNT/β-catenin pathways rather than acting as a primary driver of androgenetic alopecia susceptibility. Experimental studies demonstrate that VDR activity is required for normal postnatal hair cycling and anagen re-entry, with VDR functioning downstream or permissively alongside β-catenin–dependent programmes that regulate follicular stem cell activation and differentiation (Cianferotti et al., 2007; Pálmer et al., 2008; Demay, 2012). In dermal papilla and follicular systems, vitamin D signalling has been shown to influence hair-inductive properties, supporting biological plausibility for VDR-mediated modulation of follicular competence (Cianferotti et al., 2007; Pálmer et al., 2008). However, unlike androgen or core WNT loci, VDR has not emerged as a replicated susceptibility gene in large AGA GWAS, and clinical studies linking vitamin D status to AGA severity remain observational and non-causal. Accordingly, VDR is best viewed as a pathway-level modifier of follicular cycling capacity, with translational relevance that remains inferential and requires controlled evaluation in androgenetic alopecia.

Pathway-level and transcriptomic analyses provide the bridge from these dispersed loci to coherent biological mechanisms. Gene set enrichment and network approaches highlight that many AGA risk genes fall into androgen metabolism, canonical WNT/β-catenin signalling, TGF-β and BMP pathways, apoptosis regulation, and extracellular matrix and collagen modules, all of which are central to the control of hair follicle cycling and dermal papilla integrity (Heilmann-Heimbach et al., 2017; Michel et al., 2017; Li et al., 2024). Functional studies of bald *versus* non-bald scalp confirm that prostaglandin D_2_ (PGD_2_) and its receptor GPR44 are upregulated in balding areas and that PGD_2_ directly inhibits hair growth, while prostaglandin F_2_α analogues, acting through PTGFR, have pro-anagen effects, providing a mechanistic substrate for the associations observed at PTGDR2, PTGFR, and PTGES2 (Nieves and Garza, 2014; Francès et al., 2024). The identification of COL1A1 and other collagen-related genes, together with histological evidence of perifollicular fibrosis and microinflammation, supports an important role for extracellular matrix remodelling and low-grade immune activation in the transition from reversible miniaturisation to irreversible follicle atrophy (Martinez-Jacobo et al., 2018; Heymann, 2022; Plante et al., 2022; Francès et al., 2024). Multi-omic and Mendelian randomisation work adds a further layer by linking genetically shorter leukocyte telomere length with higher AGA risk and by showing coordinated changes in hypoxia, oxidative stress, and inflammatory gene expression in affected follicles, suggesting that telomere dynamics, metabolic stress, and immune signalling modulate the long-term resilience of the follicular stem-cell compartment (Goldberg, 2023; Liu et al., 2025b).

Prostaglandin signalling represents one of the consistently supported non-androgenic biological axes implicated in androgenetic alopecia, with convergent evidence from genetic, transcriptomic, and functional studies. Analyses of balding *versus* non-balding human scalp have repeatedly demonstrated increased expression of prostaglandin D_2_–related enzymes and receptors, including PTGDS and PTGDR2, indicating a shift towards inhibitory prostaglandin signalling in affected follicles (Garza et al., 2012; Nieves and Garza, 2014). Functional experiments have shown that elevated PGD_2_ suppresses hair shaft elongation and promotes premature catagen, providing a mechanistic explanation for the association between altered prostaglandin balance and follicular miniaturisation. In contrast, prostaglandin F_2_α signalling through PTGFR is associated with pro-anagen effects, and pharmacological analogues such as latanoprost have demonstrated modest follicular enlargement in small human studies (Blume-Peytavi et al., 2012; Suchonwanit et al., 2019). Importantly, genetic associations at PTGDR2, PTGFR, and PTGES2, together with RNA-expression differences observed in balding scalp, support the interpretation that inter-individual variation in prostaglandin synthesis and receptor signalling contributes to heterogeneity in disease progression and treatment response. While the clinical efficacy of prostaglandin-directed interventions in androgenetic alopecia remains limited and requires further validation, the prostaglandin pathway emerges as a biologically coherent and genetically supported component of AGA pathophysiology.

Clinically, this polygenic and pathway-integrated architecture provides a mechanistic explanation for the marked heterogeneity in age at onset, pattern, and progression rate of AGA, as well as for differences in comorbidity profiles and treatment responsiveness. Individuals who carry high-risk allele combinations at AR, EDA2R, and androgen-metabolism genes are likely to exhibit a lower threshold for DHT-driven catagen induction and earlier onset of miniaturisation in androgen-sensitive scalp regions, whereas additional risk alleles in WNT and morphogenetic genes may weaken anagen initiation and shorten the duration of the growth phase even when systemic androgen exposure is comparable (Li et al., 2012; Heilmann-Heimbach et al., 2017; Henne et al., 2023). Variants that affect prostaglandin synthesis and receptor signalling, vascular tone, and collagen architecture are expected to influence the quality of the follicular microenvironment, modulating angiogenesis, nutrient supply, and the balance between regenerative and fibrotic responses, which in turn may accelerate or slow the transition from miniaturised but viable follicles to scars and atrophic remnants (Garza et al., 2012; Păun et al., 2023; Francès et al., 2024). Polygenic scores derived from large GWAS already stratify men into groups with very different lifetime risks and can, in principle, be integrated with rare variant data and environmental factors to refine prognostic models (Heilmann-Heimbach et al., 2017; Pirastu et al., 2017; Henne et al., 2023). At the same time, the localisation of key risk variants in druggable pathways, such as AR and SRD5A1/2 for androgen modulation and PTGFR or PTGDR2 for prostaglandin signalling, directly links susceptibility architecture with pathways that are targeted, or could be targeted, by current and emerging therapies, thereby providing the conceptual basis for pharmacogenetic approaches that aim to match patients with the interventions most likely to be effective in their specific genetic context (Garza et al., 2012; Francès et al., 2024; Liu et al., 2025b).

## 3 Pharmacogenetic insights from clinical cohorts

**FIGURE 1:**
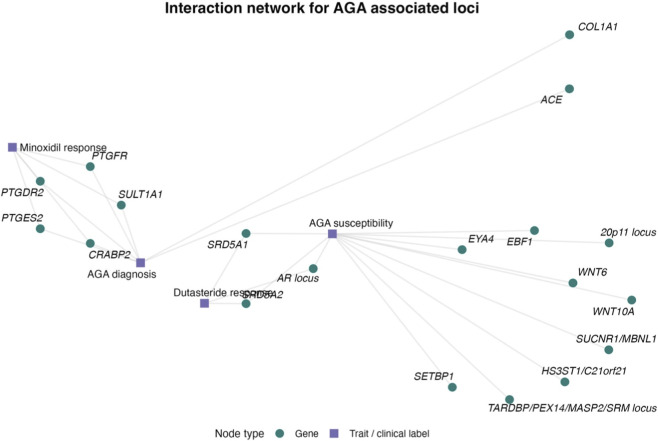
Interaction network for AGA-associated loci The network shows the relationships between androgenetic alopecia (AGA) susceptibility loci and pharmacogenetically relevant genes identified across the combined cohorts. The layout brings together functional modules that summarise key biological processes implicated in AGA. The androgen-metabolism cluster, comprising *SRD5A1* and *SRD5A2*, appears as a coherent subnetwork reflecting their shared role in dihydrotestosterone biosynthesis and relevance to dutasteride responsiveness. A second, prostaglandin-related module groups *PTGFR*, *PTGDR2*, *PTGES2*, *CRABP2*, and *SULT1A1*, which collectively influence prostaglandin balance, retinoic-acid handling, and minoxidil activation. Key susceptibility loci annotated to genes such as *AR*, *WNT10A*, *WNT6*, *EBF1*, *EYA4*, *HS3ST1/C21orf21*, *SUCNR1/MBNL1*, and the 20p11 region cluster together, highlighting convergent involvement in follicular cycling and stem-cell regulation. The network illustrates how susceptibility architecture and drug-response loci intersect, supporting hypothesis-generating and patient-stratification applications of pharmacogenetics in AGA.

Across the combined datasets, including our large-scale association study with 26,607 participants and the pharmacogenetic cohort of 252 individuals (Francès et al., 2024; Gaboardi et al., 2025), a coherent set of susceptibility and treatment-relevant *loci* emerges, reinforcing the highly polygenic nature of AGA and supporting the integration of genetics into therapeutic decision-making. The strongest signals consistently map to the AR locus, which remains the dominant contributor to male-pattern hair loss heritability (Heilmann-Heimbach et al., 2017; Henne et al., 2023). Additional replicated loci include WNT10A, WNT6, EBF1, EYA4, the 20p11 region, and the TARDBP/PEX14/MASP2/SRM cluster, which together implicate pathways governing hair-follicle morphogenesis, progenitor cell maintenance, and tissue remodelling (Yap et al., 2018a; Liu et al., 2025a). The pharmacogenetic cohort refines this architecture by identifying treatment-relevant variants in SRD5A1, SRD5A2, PTGES2, PTGFR, PTGDR2, CRABP2, SULT1A1, ACE, and COL1A1, several of which modulate responsiveness to minoxidil, finasteride, or dutasteride, thereby linking genetic predisposition not only to disease susceptibility but also to differential therapeutic efficacy (Francès et al., 2024; de Souza, 2025; Gaboardi et al., 2025). Together, these datasets allow the construction of a mechanistic and clinically interpretable framework that integrates disease biology with drug-response variability. Figure 1 illustrates these relationships as functional modules, demonstrating how susceptibility genes and pharmacogenetic markers cluster into biologically meaningful subnetworks.

**FIGURE 2:**
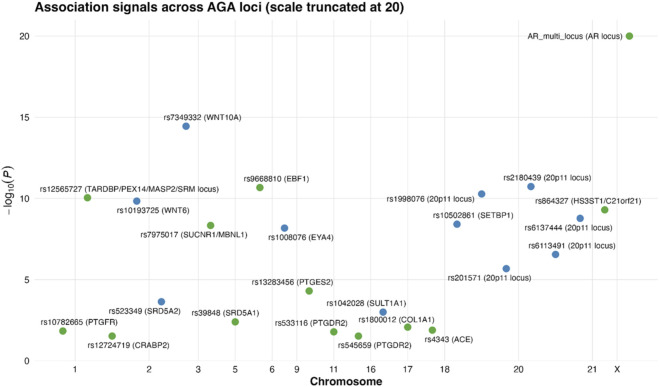
Manhattan-style plot of association signals across AGA loci. The figure displays the distribution of the most relevant AGA-associated variants across chromosomes, with p-values expressed as −log10(P) and truncated at 20 to allow visual comparison between loci. The AR multi-locus signal remains the strongest association, far exceeding genome-wide significance, but the truncation reveals the substantial evidence supporting other loci. Variants in WNT10A (rs7349332), WNT6 (rs10193725), EBF1 (rs9668810), EYA4 (rs1008076), the 20p11 region (rs1998076, rs2180439, rs201571, rs6137444), and the TARDBP/PEX14/MASP2/SRM locus (rs12565727) all reach robust significance, reflecting their repeated identification across independent studies. Variants with pharmacogenetic relevance also show consistent signals, including SRD5A1 (rs39848), SRD5A2 (rs523349), SULT1A1 (rs1042028), PTGES2 (rs13283456), PTGFR (rs10782665), PTGDR2 (rs545659/rs533116), and ACE (rs4343). Together, the plot integrates susceptibility, mechanistic, and treatment-response variants into a single view, demonstrating that AGA genetic risk is polygenic and mechanistically coherent.

**FIGURE 3:**
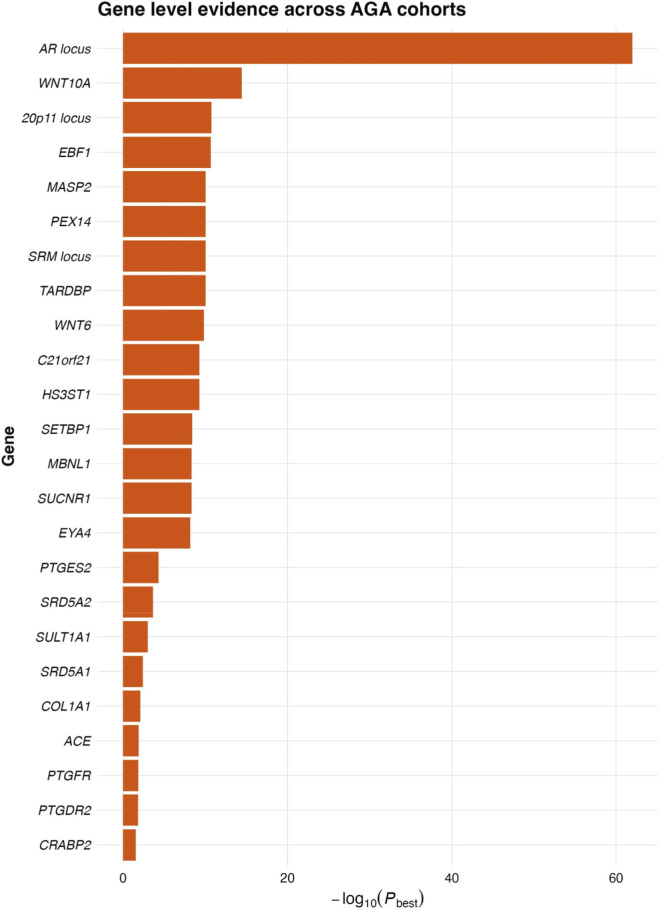
Gene-level evidence across AGA cohorts. This barplot summarises the minimum p-value observed for each gene across all included cohorts, providing a gene-centric view of the association strength. As expected, the AR locus shows the strongest evidence (−log10(P-best) > 60), consistent with its fundamental role in androgen signalling. A second tier of highly supported genes includes WNT10A, the 20p11 locus, EBF1, MASP2, PEX14, and the SRM locus, all reaching −log10(P_best) values above 10. Genes with established pharmacogenetic importance, including SRD5A1, SRD5A2, PTGES2, PTGFR, PTGDR2, SULT1A1, and ACE, also display significant but more moderate evidence, reflecting their documented yet narrower effect sizes. This gene-level synthesis highlights how major susceptibility loci and actionable pharmacogenetic targets coexist within the AGA architecture, reinforcing the rationale for integrating genetic markers into therapeutic stratification (Garza et al., 2012; Nieves and Garza, 2014; Heilmann-Heimbach et al., 2017; de Souza, 2025; Gaboardi et al., 2025).

The interaction patterns and pathway clustering shown in Figures 1–3 reflect convergence of genetic and pharmacogenetic signals across independent association studies and targeted cohorts, rather than definitive causal relationships. In this context, “co-occurrence across studies” refers to the repeated identification of variants or genes mapping to shared biological pathways involved in androgen signalling, follicular cycling, prostaglandin metabolism, and extracellular matrix regulation, as reported in genome-wide association studies and pharmacogenetic analyses (Heilmann-Heimbach et al., 2017; Henne et al., 2023; Francès et al., 2024; Gaboardi et al., 2025). While such convergence supports biological plausibility and mechanistic coherence, independent replication, ancestry-diverse validation, and prospective evaluation are required to define effect sizes and clinical decision thresholds. Accordingly, the present framework should be interpreted as hypothesis-generating and mechanism-informing, rather than as a validated clinical decision model.

For the purpose of constructing the gene-level interaction view in Figure 1, GWAS signals were represented using the gene annotations assigned in the source genome-wide association and follow-up studies, together with pharmacogenetic candidate genes evaluated directly in treatment-response cohorts. Because many MPHL-associated variants are noncoding, locus-to-gene assignments in this review should be interpreted as pragmatic mappings used for biological summarisation rather than definitive identification of causal effector genes. We did not perform new fine-mapping, colocalisation, or transcriptome-wide prioritisation analyses within this review, and chromatin interaction resources were not systematically integrated. Consistent with current post-GWAS understanding, multi-gene loci such as 20p11 remain incompletely resolved, and the network should therefore be interpreted as pathway-level synthesis of implicated loci and candidate genes rather than a causal regulatory map (Hillmer et al., 2008; Heilmann-Heimbach et al., 2017; Henne et al., 2023; Francès et al., 2024; Gaboardi et al., 2025).

The integration of genetic interactions provides further insight into how these loci modulate both disease mechanisms and treatment outcomes. Variants within the androgen metabolism cluster (SRD5A1 and SRD5A2), prostaglandin and retinoid signalling (PTGES2, PTGFR, PTGDR2, CRABP2, SULT1A1), and WNT-driven follicular programmes (WNT10A, WNT6) exhibit patterns of co-occurrence across studies, reinforcing the interplay between androgen signalling, immune-modulatory lipids, and the epithelial stem-cell niche (Henne et al., 2023). These clusters map closely onto known pharmacological pathways. SRD5A1 and SRD5A2 determine the activity of type 1 and type 2 5-alpha-reductase and thereby the expected response to finasteride or dutasteride, while prostaglandin-related genes modulate pathways targeted by latanoprost, cetirizine, and emerging prostaglandin-modifying actives. SULT1A1 affects the conversion of minoxidil into its active sulphated form, providing a mechanistic basis for predicting minoxidil responsiveness. These interactions are captured visually in Figure 1 (gene and pathway network), Figure 2 (Manhattan plot of association signals across the genome), and Figure 3 (gene-level significance summary), which together highlight how susceptibility and pharmacogenetic markers intersect at both pathway and therapeutic levels. The Manhattan plot underscores this structure by displaying convergent significance for WNT signalling, androgen signalling, and prostaglandin metabolism loci, while the gene-level summary identifies the relative weight of each pathway within the genetic architecture.

Taken together, these findings illustrate how genetic data from both large-scale susceptibility studies and targeted pharmacogenetic cohorts converge to form a unified model of AGA biology with immediate translational relevance. The replicated involvement of AR, WNT10A, WNT6, and the 20p11 region highlights stable, evolutionarily conserved drivers of hair-follicle miniaturisation (Yap et al., 2018a, 2018b; Henne et al., 2023) while the identification of treatment-modifying variants in SRD5A1, SRD5A2, PTGES2, PTGFR, PTGDR2, SULT1A1, and ACE demonstrates that the same architecture contains clinically actionable nodes. These datasets support the application of pharmacogenetics to stratify patients for minoxidil, finasteride, dutasteride, or prostaglandin-pathway–targeting agents, and they provide mechanistic hypotheses for emerging therapies, including WNT agonists and RNA-based androgen receptor suppression. The figures described above integrate these insights visually. Figure 1 demonstrates how susceptibility and treatment-response loci cluster into biologically coherent modules. Figure 2 summarises genome-wide association signals across implicated genes, and Figure 3 contextualises gene-level strength of evidence. Together, they support a framework in which genetic profiling refines mechanistic understanding, enhances prediction of therapeutic outcomes, and guides the development of personalised regimens for the management of AGA (Pirastu et al., 2017; Yap et al., 2018a, 2018b; Goldberg, 2023).

## 4 Genetic modulation of drug activity

### 4.1 Minoxidil and metabolism considerations

Topical minoxidil requires intrafollicular bioactivation to minoxidil sulphate by sulphotransferase enzymes, particularly SULT1A1, which provides a mechanistic rationale for inter-individual variability in clinical response. While the enzymatic activation of minoxidil *via* sulphotransferases represents a well-established and necessary pharmacokinetic step for efficacy, several downstream pharmacodynamic mechanisms, including its effects on follicular signalling, ion channels, and growth factor modulation, remain incompletely defined and continue to be refined. Although the pharmacodynamic effect of topical therapy depends primarily on SULT1A1 activity within the follicular outer root sheath, this enzyme, and related SULT isoforms, are expressed systemically, which means that minoxidil administered orally may undergo efficient activation outside the follicular microenvironment. This distinction helps explain why topical minoxidil is more dependent on local sulfonation capacity, whereas oral minoxidil benefits from broader systemic activation that may partially overcome reduced follicular SULT1A1 activity. Early work using plucked hair follicles demonstrated that responders exhibit substantially higher SULT1A1 activity than non-responders, and that simple enzymatic assays performed on a minimal number of follicles may predict treatment success with clinically meaningful accuracy (Goren et al., 2014, 2015). Subsequent pharmacogenetic studies identified functional SULT1A1 variants associated with altered enzyme stability and expression, complementing observational data showing that reduced activity correlates with diminished benefit from topical minoxidil (Ramos et al., 2020; Li et al., 2024). Findings from a prospective study of low dose oral minoxidil (LDOM) showed that baseline follicular SULT1A1 activity stratified outcomes, although in this context individuals with low activity exhibited greater improvement, possibly reflecting compensation by systemic exposure or engagement of alternative metabolic sites (Jimenez-Cauhe et al., 2025). A recent genetic study further confirmed that variation in SULT1A1 contributes to treatment heterogeneity by influencing predicted enzymatic output rather than allele specific effects (Gaboardi et al., 2025). Although some of these enzymatic and pharmacogenetic observations were derived from studies in women with female pattern hair loss, they are cited here as mechanistic evidence and are interpreted in this review in the context of men with AGA. Taken together, these data justify integrating follicular SULT1A1 activity with genetic determinants to support decisions between topical and oral minoxidil, recognising that recent observations on LDOM dose response and concentration remain exploratory and should not yet inform clinical dose adjustment (Goren et al., 2014; Jimenez-Cauhe et al., 2025).

### 4.2 5-Alpha-Reductase inhibitors

Type 1 and type 2 5α-reductase, encoded by SRD5A1 and SRD5A2, catalyse the conversion of testosterone to dihydrotestosterone (DHT) in a tissue dependent manner and are the principal pharmacological targets of finasteride and dutasteride, with finasteride acting selectively on type 2 and dutasteride inhibiting both isoenzymes. Foundational work in prostate biology demonstrated that common SRD5A2 variants, including V89L and A49T, reduce enzymatic activity and modulate intraprostatic DHT, influencing the risk of prostate cancer, benign prostatic hyperplasia (BPH) severity, and biochemical sensitivity to finasteride (Makridakis et al., 1999; Makridakis and Reichardt, 2005). SRD5A1 expression and regulatory variation likewise alter DHT generation in skin and scalp (Hoffmann, 2002). Contemporary AGA GWAS repeatedly identify both SRD5A1 and SRD5A2 as susceptibility *loci*, while a pharmacogenetic-focused study and others show that variants associated with increased predicted enzymatic output tend to correlate with superior clinical response to 5α-reductase inhibitors, with lower activity haplotypes showing attenuated benefit (Heilmann-Heimbach et al., 2017; Yap et al., 2018a; Gaboardi et al., 2025). This mirrors treatment patterns in BPH, where patients with high 5α-reductase activity show more pronounced prostate volume reduction after finasteride and dutasteride (Djavan et al., 2004). These converging lines of evidence indicate that the intrinsic balance between type 1 and type 2 isoenzyme activity influences treatment outcomes in AGA and support a role for SRD5A1/SRD5A2 informed decision making when selecting between finasteride and dutasteride or when identifying candidates who may require alternative therapies (Makridakis and Reichardt, 2005; Yap et al., 2018a, 2018b).

In addition to pharmacodynamic differences at the enzyme level, inter-individual variability in response to oral 5α-reductase inhibitors may also reflect systemic pharmacokinetic factors. Both finasteride and dutasteride undergo hepatic metabolism predominantly *via* CYP3A, with dutasteride characterised by a markedly prolonged terminal half-life, which may contribute to variability in systemic exposure, drug–drug interaction sensitivity, and time-dependent response profiles among patients receiving oral therapy (ROEHRBORN et al., 2004; Makridakis and Reichardt, 2005; Nickel et al., 2011).

### 4.3 Prostaglandin pathways

Prostaglandin biology adds another genetically influenced axis relevant to AGA pathophysiology and treatment. Balding scalp shows markedly elevated prostaglandin D_2_ (PGD_2_), driven by increased PTGDS expression, and exogenous PGD_2_ inhibits follicular growth through PTGDR2, triggering premature catagen and suppressing anagen maintenance (Garza et al., 2012). Conversely, prostaglandin F_2_α (PGF_2_α) analogues such as latanoprost and bimatoprost prolong anagen and stimulate follicular enlargement, which is clinically exploited in eyelash and eyebrow enhancement and supported by small proof-of-concept trials in AGA showing modest density gains (Blume-Peytavi et al., 2012; Suchonwanit et al., 2019). Several AGA genetic studies and our internal pharmacogenetic analysis identify variation in PTGFR, PTGDR2, PTGES2, and related pathway genes, reinforcing that the balance between inhibitory PGD_2_ signalling and pro-growth PGF_2_α signalling varies between individuals and may influence sensitivity to treatments that modulate this axis (Sivas, 2015; Cosmetics, 2024; Cosmetics, 2023). The convergence of genetic, biochemical, and clinical data supports the prostaglandin pathway as a mechanistically coherent target for therapeutic intervention and raises the possibility that prostaglandin receptor genotypes may aid in predicting benefit from agents such as PGF_2_α analogues, topical cetirizine, or combination regimens that indirectly lower PGD_2_ signalling (Garza et al., 2012; Francès et al., 2024).

### 4.4 Emerging targets

Beyond established pharmacological pathways, several emerging genetic targets link AGA susceptibility with follicular energy metabolism, hormonal signalling, and regenerative potential. IGF1R signalling supports dermal papilla cell survival and matrix keratinocyte proliferation, and inhibition of IGF1R impairs anagen maintenance in experimental systems, consistent with recent GWAS signals implicating IGF1R in AGA (Upton et al., 2015; Chew et al., 2016; Henne et al., 2023). WNT10A, a canonical Wnt ligand repeatedly identified in AGA association studies, regulates hair follicle morphogenesis and stem cell activation, with loss-of-function mutations causing ectodermal dysplasia and hypotrichosis, while common regulatory variants likely modulate follicular regeneration capacity in adults (Heilmann et al., 2013; Heilmann-Heimbach et al., 2017; Liu et al., 2025b). PPARGC1A (PGC1α), a central regulator of mitochondrial biogenesis and oxidative metabolism, influences follicular stem cell quiescence and reactive oxygen species handling, providing a metabolic framework that may interact with treatment response in miniaturised follicles (Geyfman et al., 2012; Li et al., 2012). Finally, prolactin receptor (PRLR) signalling has emerged as a compelling candidate: prolactin and PRLR modulate human follicular cycling, with excess prolactin promoting regression and receptor blockade partially reversing this effect (Craven et al., 2006; Foitzik et al., 2006; Langan et al., 2010). In cattle, truncating PRLR mutations produce the slick hair phenotype, characterised by short, sparse, thermotolerant fibres, demonstrating that relatively small changes in receptor function may profoundly alter hair architecture (Littlejohn et al., 2014; Porto-Neto et al., 2018; Sosa et al., 2021). Together, these mechanistic insights and early preclinical developments, including animal and *ex vivo* evidence for PRLR involvement, position IGF1R, WNT10A, PGC1α, and PRLR as genetically anchored candidates for future AGA therapies that integrate metabolic optimisation, hormonal modulation, and regenerative approaches. ((Littlejohn et al., 2014; Grymowicz et al., 2020; Littlejohn et al., 2014). This comparative phenotype illustrates the sensitivity of hair architecture to PRLR signalling across species; however, the cattle slick-hair model reflects a distinct developmental and thermoregulatory context, and does not imply a direct or magnitude-equivalent therapeutic effect of PRLR modulation in adult human scalp follicles.

JAK–STAT pathway modulation represents a pipeline-adjacent therapeutic strategy in androgenetic alopecia that is mechanistically relevant at the level of inflammatory signalling and immune-mediated follicular stress, rather than a primary genetic driver of disease susceptibility. Small-molecule JAK inhibitors have demonstrated robust efficacy in alopecia areata, where immune privilege collapse and cytotoxic lymphocyte activity are central, but their role in male pattern hair loss appears more indirect and context-dependent (Betz et al., 2015; Ungar et al., 2025). Experimental and transcriptomic studies indicate that low-grade perifollicular inflammation and cytokine signalling may contribute to disease progression in subsets of patients with androgenetic alopecia; however, genetic association data do not currently support JAK–STAT genes as core replicated loci comparable to AR or WNT10A/WNT6. Accordingly, JAK–STAT modulation is best viewed as a pharmacological adjunct that may interact with inflammatory microenvironmental features or comorbid scalp conditions, rather than as a genetically anchored target within the primary pharmacogenetic framework of androgenetic alopecia (Liu et al., 2025b; Ungar et al., 2025).

## 5 Clinical translation and personalised therapy

Topical minoxidil requires bioactivation to minoxidil sulphate by sulphotransferase enzymes, most prominently SULT1A1, which are expressed in the outer root sheath but also systemically across multiple tissues, providing a mechanistic explanation for inter-individual variability in therapeutic outcome (Messenger and Rundegren, 2004; Goren and Naccarato, 2018). Early studies analysing plucked follicles demonstrated that responders exhibit significantly higher intrafollicular SULT1A1 activity, and that simple enzymatic assays performed on a small number of follicles predict clinical benefit with meaningful sensitivity and specificity, establishing SULT1A1 activity as a limiting determinant of pharmacodynamic effect in the scalp (Goren et al., 2014, 2015; Pietrauszka and Bergler-Czop, 2022). Pharmacogenetic data further support this framework, with several studies reporting that functional SULT1A1 variants associated with reduced enzymatic stability or transcription correlate with lower response rates to topical minoxidil, in agreement with broader pharmacogenomic observations in other drug classes (Liu et al., 2014; Ramos et al., 2020) The relevance of this pathway extends to oral therapy: recent prospective work on low dose oral minoxidil showed that baseline follicular SULT1A1 activity stratified treatment response, although in this context lower activity predicted greater improvement, a finding that may reflect additional systemic sites of sulfonation and the influence of maintained plasma exposure (Jimenez-Cauhe et al., 2025). A multicentre association study further demonstrated that polygenic determinants underlying SULT1A1 activity contribute to therapeutic heterogeneity without relying on single-variant effects (Francès et al., 2024). These convergent observations justify the integration of SULT1A1-based functional assays with genotypic information to refine the prediction of minoxidil responders and guide the selection between topical and oral regimens, while recognising that emerging insights into dose–response dynamics for oral minoxidil remain investigational and require further validation (Olsen et al., 2002, 2007; Lucky et al., 2004; Jimenez-Cauhe et al., 2025).

Finasteride and dutasteride exert their therapeutic actions by inhibiting type 2 and dual type 1/type 2 5α-reductase activity, respectively, thereby reducing the conversion of testosterone to dihydrotestosterone in scalp tissue. Although most mechanistic understanding originates from prostate and dermatological studies, several lines of evidence support a clinically meaningful role for SRD5A1 and SRD5A2 variation in modulating treatment efficacy. Functional work has shown that common SRD5A2 missense variants, including V89L and A49T, alter enzyme kinetics and influence circulating and intraprostatic dihydrotestosterone concentrations, providing a biochemical basis for variable response to 5α-reductase inhibition (Makridakis et al., 1999; Makridakis and Reichardt, 2005). Parallel observations indicate that SRD5A1 transcriptional regulators influence local androgen metabolism in the skin and may contribute to regional sensitivity in androgenetic alopecia (Hoffmann, 2002). Large trials in benign prostatic hyperplasia, such as the CombAT study, established that dutasteride suppresses dihydrotestosterone more extensively than finasteride, confirming the functional relevance of type 1 inhibition *in vivo* (Roehrborn et al., 2004; Nickel et al., 2011; Unger et al., 2016). Scalp-focused genetic association studies now implicate both SRD5A1 and SRD5A2 loci in androgenetic alopecia susceptibility and suggest that alleles associated with higher predicted enzymatic output are linked with improved response to 5α-reductase inhibitors, whereas reduced-activity haplotypes show attenuated benefit (Mononen et al., 2001; Giwercman et al., 2005; Heilmann-Heimbach et al., 2017; Yap et al., 2018a) When synthesised, these findings support a model in which SRD5A1/SRD5A2 genotypes, interpreted with knowledge of finasteride and dutasteride pharmacology, may help prioritise therapy selection, particularly in individuals with suboptimal response to finasteride or in those whose genetic background suggests reduced sensitivity to selective type 2 inhibition (Roehrborn et al., 2004; Heilmann-Heimbach et al., 2017).

Prostaglandin-based therapies, particularly latanoprost and bimatoprost, represent a potential adjunct or alternative for patients who do not respond adequately to minoxidil, although the current clinical evidence remains modest and exploratory. The rationale builds on the clear dichotomy between prostaglandin D_2_, which is elevated in balding scalp and suppresses hair shaft elongation through PTGDR2 signalling, and prostaglandin F_2_α analogues, which prolong anagen and stimulate follicular enlargement (Garza et al., 2012; Yao and Narumiya, 2019). Early human work demonstrated that topical latanoprost applied to the scalp increased hair density and thickened miniaturised follicles over several months, paralleling its known effects in stimulating eyelash and eyebrow growth (Tosti et al., 2004; Blume-Peytavi et al., 2012). Case reports and mechanistic studies also describe hypertrichosis induced by latanoprost and bimatoprost, reinforcing the biological plausibility of FP-receptor mediated follicular stimulation (Christiansen et al., 2004; Wester et al., 2010). Emerging pharmacogenetic analyses indicate that variants in PTGFR and PTGDR2 modify prostaglandin balance and may shape the magnitude of response to prostaglandin-targeted therapies or cetirizine-based regimens (Francès et al., 2024; Gaboardi et al., 2025). While the evidence does not yet support routine use of latanoprost or bimatoprost for androgenetic alopecia, these observations suggest that prostaglandin pathway profiling may help identify individuals more likely to benefit from such treatments, particularly where minoxidil response is absent or limited (Tosti et al., 2004; Blume-Peytavi et al., 2012).

Together, these therapeutic examples illustrate how genetic and functional markers may be incorporated into decision making frameworks for male pattern hair loss. SULT1A1 activity and genotype refine the prediction of minoxidil responsiveness and guide the choice between topical and oral modalities, SRD5A1/SRD5A2 variation contextualises the relative likelihood of benefit from finasteride *versus* dutasteride, and prostaglandin pathway markers help delineate scenarios in which latanoprost or bimatoprost may offer supplementary value. Although many of these applications remain in development, the convergence of genomic association data, functional enzymology, and clinical pharmacology demonstrates that personalised therapeutic strategies are feasible and scientifically grounded, supporting a progressive shift from empirical selection to genetically informed treatment optimisation in male pattern hair loss (ROEHRBORN et al., 2004; Heilmann-Heimbach et al., 2017; Jimenez-Cauhe et al., 2025).

## 6 Genetic pathways and novel therapies

Novel regenerative and nucleic acid based approaches for male pattern hair loss exploit the same genetic pathways that underlie disease susceptibility, particularly AR signalling and follicular microenvironmental homeostasis. Small interfering RNAs (siRNAs) and related constructs target AR mRNA to reduce receptor abundance in dermal papilla cells, thereby lowering the transcriptional response to dihydrotestosterone and attenuating miniaturisation. Early oncology work showed that systemic AR-directed siRNA suppresses tumour growth, angiogenesis, and proliferation in castration-resistant prostate cancer, demonstrating that AR remains a drug-related target even in resistant states (Compagno et al., 2007). In AGA specific models, several formats refine this principle. Self-assembled micelle inhibitory RNA (SAMiRNA-AR68) incorporates siRNA into a nanoparticle that penetrates the outer root sheath and dermal papilla while avoiding innate immune activation (Yun et al., 2022; Moon et al., 2023). Asymmetric siRNA with a cholesterol-conjugated passenger strand (cp-asiAR) is designed for passive uptake into skin and sustained dermal retention, where it downregulates AR and dampens downstream inflammatory and WNT antagonistic mediators such as IL-6, TGF-β1, and DKK1 (Moon et al., 2023). UTR-targeting siRNAs such as AR-27 E-Chol follow a similar logic, focusing on conserved non-coding regions to maximise cross-species translation from preclinical mouse models to humans and to reduce the likelihood of resistance through coding sequence variation (Moon et al., 2023; Feng et al., 2025). In parallel, regenerative strategies based on mesenchymal stem cells and their secretome aim to restore hair follicle cycling through paracrine modulation of inflammation, angiogenesis, extracellular matrix, and stem-cell niche function. Adipose derived stem cells (ASCs) and adipose tissue derived mesenchymal stem cell secretome (AT-MSC secretome) release extracellular vesicles, growth factors, and cytokines that exert anti inflammatory, pro-angiogenic, and anti fibrotic effects, promote keratinocyte and fibroblast proliferation, and support re-epithelialisation and hair follicle regeneration, suggesting a cell-free route to influence WNT, TGF-β, and vascular pathways already implicated genetically in AGA in men (Bellani et al., 2025; Liu et al., 2025a; Sendera et al., 2025).

It should be noted that much of the mechanistic efficacy data for AR-silencing constructs derives from murine models and *ex vivo* human scalp systems, and that while early clinical trials demonstrate biological activity, preclinical potency does not necessarily translate into proportional clinical benefit in human scalp hair density. Clinical and preclinical outcome data from these modalities remain heterogeneous but provide convergent proof of concept. In DHT-induced mouse models of AGA, AR-27 E-Chol and cp-asiAR both stimulate dorsal hair regrowth, increase the proportion of follicles in anagen, and reduce cutaneous AR expression, with cp-asiAR additionally reversing DHT-induced telogen transition in *ex vivo* human scalp and preserving hair bulb diameter without measurable systemic exposure or dermal papilla cytotoxicity (Yun et al., 2022; Moon et al., 2023; Feng et al., 2025). SAMiRNA-AR68 moves this principle into human trials. In two randomised double blind studies, weekly topical application of SAMiRNA-AR68 increased hair counts and density over 24 weeks compared with vehicle, with the higher dose regimen achieving mean gains of approximately 8 hairs/cm^2^ and subjectively appreciable improvement, while exhibiting only mild, infrequent local reactions and no cytokine-mediated immune activation (Yun et al., 2022). These effects were modest in absolute magnitude but similar to those reported for finasteride in some series, suggesting that partial AR silencing at the follicular level is clinically meaningful. Stem-cell approaches are at an earlier translational stage but display consistent biological activity. In the DHT mouse model, intradermal injection of allogenic ASCs combined with ATP improved hair regrowth outperforming controls on photographic, ImageJ, and histological assessment (Bran et al., 2025). A systematic review of 15 human studies involving 653 subjects treated with hair-follicle stem cells, ASCs, stromal vascular fraction, conditioned medium, or bone marrow derived cells found that most protocols increased hair density *versus* baseline or placebo, with generally mild adverse events but substantial heterogeneity in cell source, preparation, injection schedules, and outcome definitions (Krefft-Trzciniecka et al., 2023). AT-MSC secretome based interventions in early dermatological trials, mainly in wound healing and rejuvenation, show improved re-epithelialisation, reduced inflammation, and enhanced collagen remodelling, outcomes that are mechanistically relevant for AGA but still need direct, controlled evaluation in hair loss cohorts (Sendera et al., 2025). Although these findings support biological plausibility, most evidence for stem-cell and secretome-based interventions in androgenetic alopecia remains preclinical or exploratory, and heterogeneity in cell sources, delivery protocols, and outcome measures limits direct extrapolation to predictable clinical efficacy.

Within the framework of this review, these developments position RNA interference and stem-cell derived products as mechanistically aligned extensions of pharmacogenetically guided therapy rather than isolated experimental curiosities. AR targeted siRNA and SAMiRNA strategies directly exploit the central genetic contribution of AR to AGA susceptibility and provide a means to modulate receptor activity locally in men with MPHL, in principle complementing or partially replacing systemic 5-alpha-reductase inhibition in individuals with high AR risk allele burden or intolerance to oral agents (Li et al., 2012; Heilmann-Heimbach et al., 2017; Yun et al., 2022; Moon et al., 2023). At the same time, ASC and AT-MSC secretome approaches address microenvironmental deficits in angiogenesis, extracellular matrix quality, and low-grade inflammation that are highlighted by collagen, prostaglandin, and immune related loci in genetic and transcriptomic studies, offering a route to regenerate miniaturised but viable follicles and possibly delay irreversible fibrotic remodelling (Martinez-Jacobo et al., 2018; Francès et al., 2024; Bran et al., 2025). Together, these modalities suggest a future treatment landscape in which genomic profiling identifies men with strong AR-driven disease, prostaglandin dysregulation, or fibrotic propensity, and informs the tailored use of AR silencing constructs, conventional pharmacotherapy, and stem-cell derived regenerative products in rational combinations. However, the modest effect sizes, limited duration of follow up, and lack of standardised protocols observed to date indicate that larger, rigorously controlled trials, integrated with pharmacogenetic and multi omic stratification, are required before these RNA and stem-cell based therapies may be incorporated confidently into routine management of male pattern hair loss. Across these emerging modalities, it is essential to distinguish mechanistic proof-of-concept demonstrated in animal or *ex vivo* systems from clinically meaningful outcomes in humans, as differences in follicular architecture, disease chronicity, and tissue remodelling constrain the translation of preclinical efficacy into therapeutic magnitude.

## 7 Conclusion and future perspectives

Pharmacogenetic studies in androgenetic alopecia have moved the field from largely empirical prescribing towards a mechanistically informed framework in which variation in drug targets, metabolising enzymes, and signalling pathways explains at least part of the heterogeneity in treatment response. Current data already support clinically meaningful roles for SULT1A1 in minoxidil activation, SRD5A1 and SRD5A2 in 5α-reductase inhibitor efficacy, and prostaglandin pathway genes such as PTGES2, PTGFR, and PTGDR2 in modulating sensitivity to prostaglandin-directed therapies, alongside contributions from collagen, vascular, and retinoid-related loci (Garza et al., 2012; Heilmann-Heimbach et al., 2017; Francès et al., 2024; Gaboardi et al., 2025; Liu et al., 2025b). Future progress will depend on moving beyond single-SNP associations towards polygenic response scores, explicit SNP–SNP interaction models, and machine-learning approaches that integrate multiple loci, routes of administration, and drug classes while preserving biological interpretability. Larger, ancestry-diverse, prospectively followed cohorts, embedded in interventional studies, are required to validate these predictors, clarify effect sizes, and expand the catalogue of actionable targets, including emerging candidates such as IGF1R, WNT10A, PPARGC1A, and PRLR that link metabolic and regenerative programmes with clinical outcomes (Li et al., 2012; Henne et al., 2023; de Souza, 2025).

A clinically useful precision framework for AGA in men will also require tight integration of genetics with detailed clinical phenotyping and multi-omic profiling. High-quality epidemiological and clinical work has shown that AGA in men comprises a spectrum of patterns, ages of onset, endocrine backgrounds, and comorbidity profiles across ancestries, which must be captured systematically if genetic signals are to be translated into robust endotypes with therapeutic relevance, while distinct female hair loss phenotypes require separate consideration and are outside the scope of this review (Su and Chen, 2010; Starace et al., 2020; Liu et al., 2025b). Transcriptomic and pathway analyses of balding *versus* non-balding scalp, including datasets highlighting androgen-driven senescence, WNT suppression, and prostaglandin imbalance, already demonstrate that GWAS loci converge on coherent biological modules in dermal papilla cells, the epithelial stem-cell niche, and the perifollicular microenvironment (Leirós et al., 2012; Upton et al., 2015; Li et al., 2024; Liu et al., 2025a). Extending these efforts to incorporate longitudinal transcriptomics, proteomics, and metabolomics in patients undergoing pharmacological, regenerative, or RNA-based interventions might permit the construction of response signatures that link specific genetic backgrounds with dynamic tissue-level changes, thereby informing rational use of minoxidil, 5α-reductase inhibitors, prostaglandin analogues, platelet-rich plasma, microneedling, stem-cell-derived products, and AR-silencing constructs in defined clinical subgroups (Ersan et al., 2024; Bran et al., 2025; Feng et al., 2025; Zhi et al., 2025).

Taken together, the evidence summarised in this review indicates that AGA in men is a highly prevalent, psychosocially burdensome, yet mechanistically tractable condition in which genetic architecture, tissue biology, and treatment response are tightly interwoven (Aukerman and Jafferany, 2023; Liu et al., 2025b). Common and rare variants in androgen, WNT, prostaglandin, extracellular matrix, metabolic, and regenerative pathways refine understanding of pathophysiology and already yield a first generation of pharmacogenetic markers that may guide the choice and sequence of therapies for individual patients (Heilmann-Heimbach et al., 2017; Henne et al., 2023; Francès et al., 2024; Gaboardi et al., 2025). The next phase should focus on embedding validated genetic predictors and polygenic scores, together with multi-omic biomarkers, into the design of clinical trials for both established and emerging treatments, using genotype and pathway-defined endotypes as inclusion strata and prospective modifiers of outcome. As analytical validity, clinical utility, and cost-effectiveness are established, routine integration of pharmacogenetic profiling into hair restoration and regenerative medicine practice for male pattern hair loss is a realistic goal, with the prospect of moving from average-effect regimens towards personalised, mechanism-anchored treatment algorithms that align therapeutic intensity and modality with each patient’s biological risk and potential for follicular recovery (Li et al., 2012; Pirastu et al., 2017; Liu et al., 2025b).
